# The Organogermanium Compound 3-(trihydroxygermyl)propanoic Acid Exerts Anti-Inflammatory Effects via Adenosine-NR4A2 Signaling

**DOI:** 10.3390/ijms26062449

**Published:** 2025-03-09

**Authors:** Junya Azumi, Tomoya Takeda, Shunya Shibata, Yasuhiro Shimada, Hisashi Aso, Takashi Nakamura

**Affiliations:** 1 Research Division, Asai Germanium Research Institute Co., Ltd., Suzuranoka 3-131, Hakodate 042-0958, Hokkaido, Japan; tomo.t621@asai-ge.co.jp (T.T.); shibata@asai-ge.co.jp (S.S.); y.shimada@asai-ge.co.jp (Y.S.); hisashi.aso.a6@tohoku.ac.jp (H.A.); nakamura@asai-ge.co.jp (T.N.); 2 Laboratory of Animal Health Science, Graduate School of Agricultural Science, Tohoku University, 468-1, Aramaki Aza, Aoba, Sendai 980-8578, Miyagi, Japan

**Keywords:** 3-(trihydroxygermyl)propanoic acid (THGP), Ge-132, inflammasome inhibition, adenosine metabolism, NR4A2 nuclear receptor, cytokine suppression

## Abstract

We previously reported that 3-(trihydroxygermyl)propanoic acid (THGP) suppresses inflammasome activation in THP-1 cells following stimulation with lipopolysaccharide (LPS) and ATP (signals 1 and 2) by forming a complex with ATP, thereby inhibiting IL-1β secretion. Our findings also suggested that THGP inhibits inflammasome activation through mechanisms independent of ATP complex formation. This study investigated the anti-inflammatory effects of THGP on signal 1 (ATP-independent) of inflammasome activation. THGP suppressed NF-κB nuclear translocation in LPS-stimulated THP-1 cells, which reduced the mRNA expression of the proinflammatory cytokines TNF-α and IL-6, as well as IL-1β secretion. This mechanism was mediated by the formation of a THGP–adenosine complex, which inhibited adenosine degradation and subsequently activated adenosine–NR4A2 signaling. Thus, THGP exerts anti-inflammatory effects by forming a complex with adenosine, leading to adenosine–NR4A2 signaling pathway activation. This mechanism is distinct from the ATP-dependent pathway by which THGP was previously reported to function. By targeting both ATP-dependent and ATP-independent inflammasome activation pathways, THGP has potential as a broad-spectrum therapeutic agent for various inflammatory diseases.

## 1. Introduction

Inflammation is a biological defense mechanism that is induced by harmful stimuli such as infections, injuries, or toxins, and the innate immune system plays a central role in this defense [1]. The inflammatory response begins and progresses through the recognition and phagocytosis of pathogens by monocytes and macrophages, the secretion of inflammatory cytokines, and the polarization of macrophages toward the M1 and M2 phenotypes [2]. These processes are essential for eliminating pathogens or damaged tissues and initiating tissue repair [3]. However, chronic inflammation can lead to excessive tissue damage and contribute to the progression of various diseases, including inflammatory diseases such as rheumatic diseases, ulcerative colitis, and Crohn’s disease [4,5]. These conditions are caused by abnormal immune responses and are associated with chronic inflammation [6]. Chronic inflammation significantly impacts disease progression and exacerbation, leading to a marked decline in patients’ quality of life.

Interleukin-6 (IL-6) and tumor necrosis factor-α (TNF-α) are major cytokines that play critical roles in the inflammatory response [7]. During bacterial or viral infections, the p38 MAPK pathway becomes activated, leading to the phosphorylation and degradation of IκB and the subsequent release of NF-κB, which then translocates to the nucleus [8,9]. Once it is translocated to the nucleus, NF-κB is phosphorylated and binds to κB binding sites, promoting the transcription of proinflammatory cytokines such as IL-6 and TNF-α [10]. Unlike these cytokines, Interleukin-1β (IL-1β) secretion requires the activation of a protein complex called the inflammasome [11]. Inflammasome activation involves two signaling pathways: signal 1, where lipopolysaccharide (LPS)-induced NF-κB activation promotes IL-1β transcription, and signal 2, where adenosine triphosphate (ATP) facilitates inflammasome assembly and IL-1β secretion [12].

The organogermanium compound Ge-132 and its hydrolyzed derivative 3-(trihydroxygermyl)propanoic acid (THGP) have been reported to exert various biological effects, including antitumor and immunomodulator [13,14,15]. Additionally, THGP has been shown to form complexes with *cis*-diol structures [16]. Endogenous components containing *cis*-diol structures, such as adrenaline, L-DOPA, and ATP, interact with THGP and influence its biological activity [17,18,19].

We previously reported that THGP forms a complex with ATP in human monocyte-derived THP-1 cells, thereby attenuating signal 2 activity and suppressing inflammasome activation [17]. In that study, we examined the inhibitory effects of THGP on IL-1β secretion in experiments involving LPS combined with either ATP or BzATP, which is an agonist of the ATP receptor P2X7R. Although THGP strongly inhibited IL-1β secretion in response to ATP stimulation, it also had weak inhibitory effects when administered with BzATP, which cannot form a complex with THGP. These findings suggest that THGP may suppress inflammation through mechanisms other than ATP complex formation. Therefore, in this study, we investigated the effects of THGP on signal 1 during inflammasome activation.

In signal 1, NF-κB promotes the transcription of the nuclear receptor NR4A2. NR4A2 exerts anti-inflammatory effects by initiation mechanisms that negatively regulate NF-κB expression [20]. During inflammatory responses, extracellular ATP is sequentially degraded into adenosine diphosphate (ADP), adenosine monophosphate (AMP), and adenosine by ecto-ATPases [21]. Adenosine reportedly functions as an anti-inflammatory mediator by suppressing TNF-α secretion via the initiation of signals through its A2 receptor [22,23]. Furthermore, adenosine signaling activates NR4A2 to inhibit inflammation [20]. However, extracellular adenosine is rapidly degraded into inosine by ecto-adenosine deaminase (ADA) [24]. Previous studies have shown that THGP forms a complex with adenosine and inhibits its degradation by ADA [25]. Based on these findings, we hypothesized that THGP suppresses signal 1 inflammasome activation by activating the adenosine–NR4A2 signaling pathway through the inhibition of adenosine degradation.

This study aims to investigate how THGP modulates inflammatory responses by influencing adenosine metabolism and NR4A2 activation. THP-1 cells, a human monocytic cell line, serve as a well-established model for studying innate immune responses, including inflammasome activation and cytokine production. Upon differentiation with Phorbol 12-myristate 13-acetate (PMA), THP-1 cells exhibit macrophage-like characteristics, enabling the investigation of inflammatory pathways [26,27]. Therefore, we examine the impact of THGP on inflammasome activation and cytokine secretion in THP-1-derived macrophages. By elucidating the role of THGP in the adenosine–NR4A2 axis, we aim to provide new insights into its potential as an anti-inflammatory agent.

## 2. Results

### 2.1. THGP Suppresses the LPS-Induced Nuclear Translocation of NF-κB

**Figure 1:**
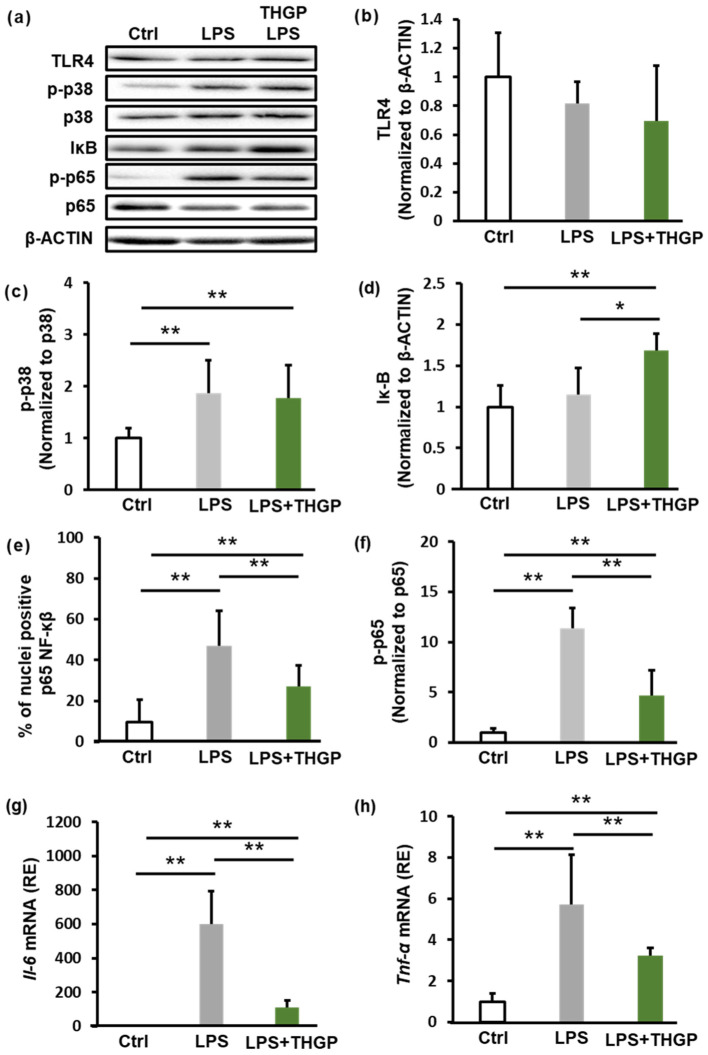
Investigation of the inhibitory effect of THGP on NF-κB signaling. THP-1 cells were seeded and treated with 1 μM PMA for differentiation. After 24 h, the cells were treated with 5 mM THGP. An additional 24 h later, 10 μg/mL LPS was added. The cells were then incubated for another 24 h, after which samples were collected. (**a**) Western blotting analysis of TLR4, p-p38, p38, IκB, p-p65, and p65 levels in THP-1 cells stimulated with LPS (10 μg/mL) and treated with THGP (5 mM). (**b**–**d**) Quantification of the band intensities from (**a**), normalized to those of β-actin. (**e**) Immunofluorescence staining showing NF-κB localization in THP-1 cells stimulated with LPS (10 μg/mL) and treated with THGP (5 mM). (**f**) Quantification of the band intensities from (**a**), normalized to those of β-actin. qPCR analysis of IL-6 (**g**) and TNF-α (**h**) mRNA expression in THP-1 cells under the same conditions. The results are shown as the means ± SDs (*n* = 6). * *p* < 0.05 and ** *p* < 0.01 compared with the control.

Using macrophage-like differentiated THP-1 cells, we investigated the p38–NF-κB signaling pathway, which is a major inflammatory signaling pathway that is activated by LPS stimulation. First, we examined the expression of TLR4, which is the receptor for LPS, and the phosphorylation of p38 (Figure 1a–c). LPS stimulation did not alter Toll-like receptor 4 (TLR4) expression, and THGP had no effect on TLR4 levels (Figure 1b). Additionally, LPS stimulation increased phosphorylated p38 MAPK (p-p38) levels by approximately two-fold; however, THGP did not suppress p-p38 activation (Figure 1c). In contrast, the nuclear translocation of NF-κB downstream of p38 was significantly affected. Initially, LPS stimulation increased the expression of IκB 1.7-fold (Figure 1d). Furthermore, LPS stimulation increased the proportion of cells with nuclear NF-κB from 10% to 47%, whereas this proportion was reduced to 27% by THGP treatment (Figure 1e). Since NF-κB functions as a transcription factor after its nuclear translocation and phosphorylation, we evaluated p-p65 levels by western blotting. LPS stimulation increased p-p65 levels by 11.3-fold, and THGP treatment suppressed this increase by approximately 37% (Figure 1f). Finally, we analyzed the mRNA expression of IL-6 and TNF-α, which are target genes of NF-κB, by RT-PCR. Compared with that of the control, LPS increased IL-6 expression by 600-fold, whereas THGP suppressed this increase to 110-fold (Figure 1g). Similarly, TNF-α expression increased 5.7-fold with LPS stimulation, and this effect was suppressed to 3.3-fold by THGP (Figure 1h). These results indicate that THGP suppresses LPS-induced inflammatory responses by inhibiting IκB degradation and NF-κB nuclear translocation. 

### 2.2. Adenosine Suppresses Inflammation

**Figure 2:**
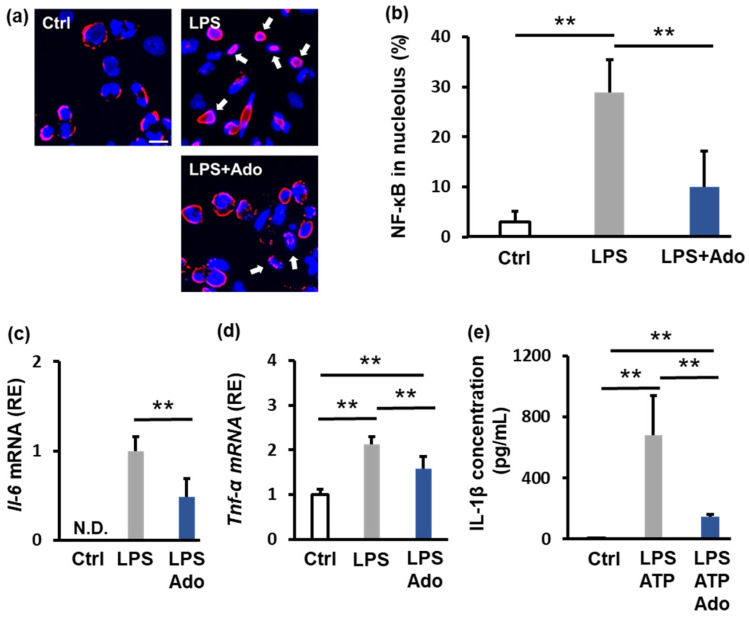
Analysis of the anti-inflammatory effects of adenosine. THP-1 cells were seeded and treated with 1 μM PMA for differentiation. After an additional 24 h, 10 μg/mL LPS and 100 μM adenosine were added. The cells were then incubated for another 24 h, after which samples were collected. For IL-1β concentration measurement, the cells were stimulated with 1 mM ATP for 2 h. (**a**) Immunofluorescence staining showing NF-κB localization in THP-1 cells stimulated with LPS (10 μg/mL) and treated with adenosine (100 μM). Red: NF-κB; blue: DAPI. The white arrows indicate NF-κB localized in the nucleus. (**b**) Quantification of the nuclear NF‒κB ratio is shown in (**a**). The results of qPCR analysis of IL-6 (**c**) and TNF-α (**d**) mRNA expression under the same conditions in (**a**). (**e**) IL-1β secretion was measured by ELISA in THP-1 cells stimulated with LPS (10 μg/mL), ATP (1 mM), and adenosine (100 μM). The results are shown as the means ± SDs (*n* = 6). and ** *p* < 0.01 compared with the control. Scale bar: 20 μm.

Adenosine is known to have anti-inflammatory properties; therefore, we examined the effect of 100 μM adenosine on NF-κB nuclear translocation. Adenosine suppressed the LPS-induced increase in nuclear localization of NF-κB from 30% to 10% (Figure 2a,b). Furthermore, adenosine suppressed the LPS-induced increase in the mRNA expression of the IL-6 and TNF-α by approximately 50% and 25%, respectively (Figure 2c,d). We also evaluated the level of secreted IL-1β. LPS and ATP stimulation increased IL-1β secretion from 7 pg/mL to 678 pg/mL; however, adenosine decreased IL-1β secretion to 184 pg/mL (Figure 2e). These findings demonstrate that adenosine effectively suppresses inflammation.

### 2.3. THGP Forms a Complex with Adenosine and Inhibits Its Degradation

**Figure 3:**
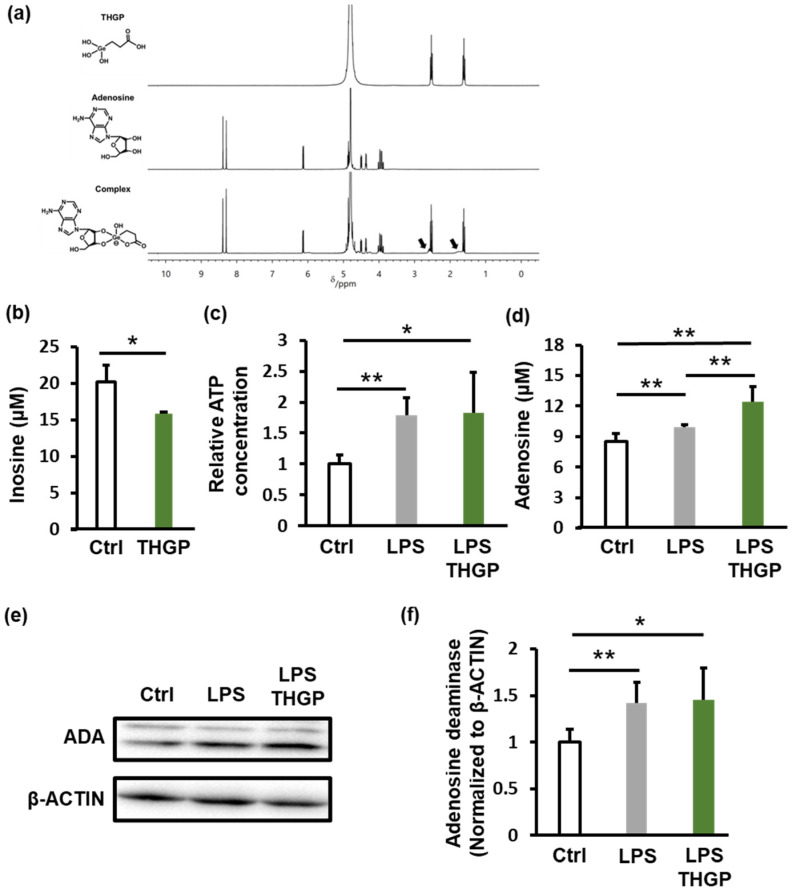
Effect of THGP on adenosine degradation. (**a**) ^1^H-NMR analysis of THGP–adenosine complex formation. The black arrows indicate signals corresponding to the complex. The left diagram shows the chemical structures of THGP, adenosine, and their complex. (**b**) Measurement of inosine levels produced by adenosine-degrading membrane proteins extracted from THP-1 cells. Quantification of ATP (**c**) and adenosine (**d**) levels in THP-1 cell culture supernatants after LPS (10 μg/mL) stimulation and THGP (5 mM) treatment. (**e**) Western blot analysis of adenosine deaminase (ADA) expression under the same conditions. (**f**) Quantification of the band intensities from (**e**), normalized to those of β-actin. The results are shown as the means ± SDs (*n* = 3‒6). * *p* < 0.05 and ** *p* < 0.01 compared with the control.

Because adenosine has a *cis*-diol structure, we investigated whether THGP forms a complex with adenosine using ^1^H-NMR analysis. When THGP and adenosine were mixed, new peaks, which were not observed for either compound alone, appeared (indicated by black arrows); these results suggested an interaction between THGP and adenosine (Figure 3a). Previous studies have shown that THGP inhibits the enzymatic degradation of adenosine through such interactions (23). To confirm this, we extracted membrane proteins from THP-1 cells and assessed their ability to degrade adenosine in the presence of THGP. The production of inosine, which is a degradation product of adenosine, decreased from 20 μM to 15 μM in the presence of THGP (Figure 3b). Next, we measured ATP and adenosine levels in culture supernatants following LPS stimulation. LPS increased ATP levels by 1.8-fold and adenosine levels from 8.5 μM to 10 μM (a 1.2-fold increase) (Figure 3c,d). Treatment with THGP did not affect ATP levels but increased adenosine levels to 12.4 μM (a 1.5-fold increase). Finally, we examined the expression of adenosine deaminase after LPS stimulation. Although LPS increased adenosine deaminase expression by 1.4-fold, THGP had no effect on its expression (Figure 3e,f). These results suggest that THGP inhibits adenosine degradation by forming a complex with adenosine, thereby increasing extracellular adenosine levels in culture supernatants.

### 2.4. THGP Suppresses Inflammation by Forming a Complex with Adenosine

**Figure 4:**
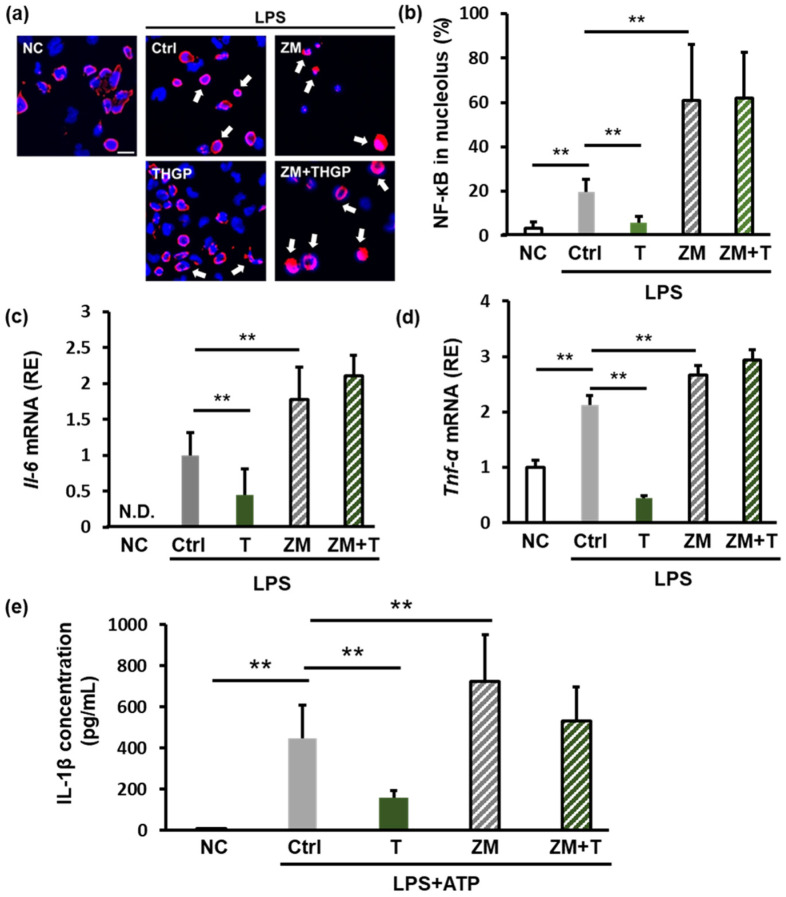
Impact of adenosine signaling inhibition on the anti-inflammatory effects of THGP. THP-1 cells were seeded and treated with 1 μM PMA for differentiation. After 24 h, the cells were treated with 5 mM THGP. An additional 24 h later, 10 μg/mL LPS and 10 μM ZM241385 were added. The cells were then incubated for another 24 h, after which samples were collected. For IL-1β concentration measurement, the cells were stimulated with 1 mM ATP for 2 h. (**a**) Images of immunofluorescence staining showing NF-κB localization (red) and nuclei (blue) in THP-1 cells treated with LPS (10 μg/mL), the A2R receptor inhibitor ZM241385 (10 μM), and THGP (5 mM). The white arrows indicate NF-κB localized in the nucleus. Scale bar: 20 μm. (**b**) Quantification of the nuclear NF‒κB ratio. qPCR analysis of IL-6 (**c**) and TNF-α (**d**) mRNA expression and ELISA measurement of IL-1β secretion (**e**) in cells stimulated with LPS (10 μg/mL) and ATP (1 mM) under the same conditions. The results are shown as the means ± SDs (*n* = 6). ** *p* < 0.01 compared with the control.

We investigated the role of adenosine signaling in the anti-inflammatory effects of THGP by administering ZM 241385, which is an antagonist of the A2 receptor (adenosine receptor). LPS stimulation increased the nuclear localization of NF-κB to 20%, and ZM 241385 further increased this localization to 60%. However, when both THGP and ZM 241385 were administered, the inhibitory effect of THGP on NF-κB nuclear translocation was reversed (Figure 4a,b). Similarly, ZM 241385 reversed the suppressive effects of THGP on LPS-induced IL-6 and TNF-α mRNA expression (Figure 4c,d). Furthermore, we assessed the levels of secreted IL-1β using an ELISA. LPS and ATP stimulation increased IL-1β secretion from 7 pg/mL to 445 pg/mL. THGP reduced this secretion to 159 pg/mL. However, ZM 241385 increased IL-1β secretion to 723 pg/mL, and cotreatment with THGP and ZM 241385 abolished the suppressive effect of THGP on IL-1β secretion (Figure 4e). These results indicate that the anti-inflammatory effects of THGP are mediated by adenosine signaling. Thus, by forming a complex with adenosine, THGP inhibits adenosine degradation and suppresses inflammation.

### 2.5. THGP Increases NR4A2 Expression

**Figure 5:**
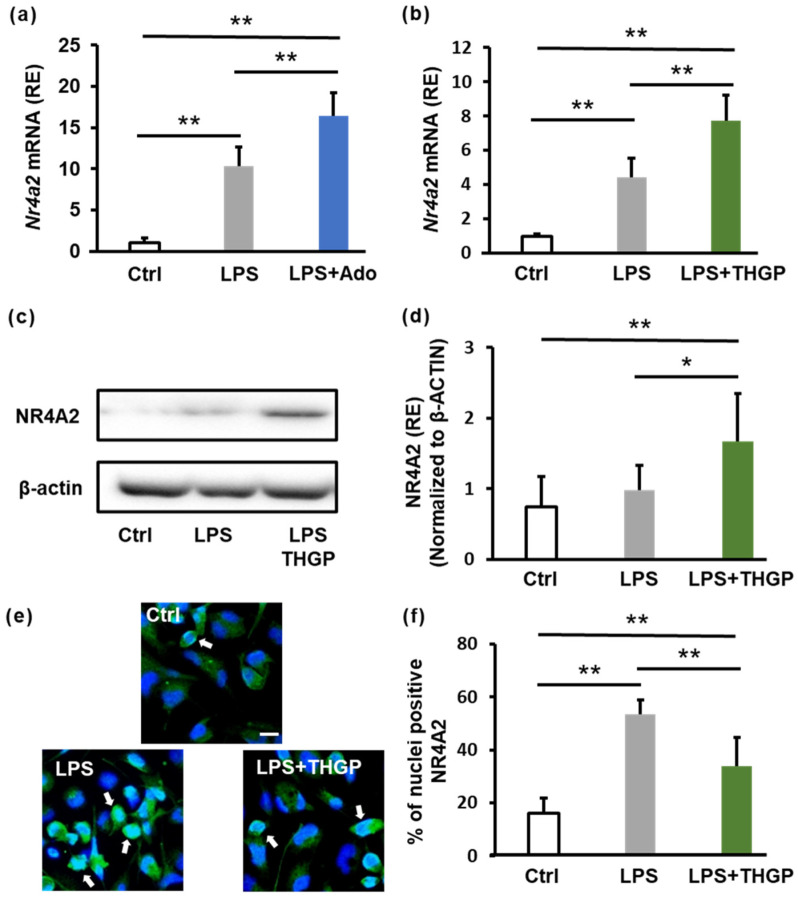
Changes in NR4A2 expression induced by adenosine or THGP. THP-1 cells were seeded and treated with 1 μM PMA for differentiation. After 24 h, the cells were treated with 5 mM THGP. An additional 24 h later, 10 μg/mL LPS was added. The cells were then incubated for another 24 h, after which samples were collected. (**a**) qPCR analysis of NR4A2 mRNA expression in THP-1 cells treated with LPS (10 μg/mL) and adenosine (100 μM). (**b**) Similar analysis after THGP (5 mM) treatment. (**c**) Western blot analysis of NR4A2 protein expression following THGP treatment. (**d**) Quantification of the band intensities from (**c**) normalized to those of β-actin. (**e**) Images of immunofluorescence staining showing NR4A2 localization (green) and nuclei (blue). Scale bar: 20 μm. The white arrows indicate NR4A2 localized in the nucleus. (**f**) Quantification of the nuclear NR4A2 ratio. The results are shown as the means ± SDs (*n* = 6). * *p* < 0.05 and ** *p* < 0.01 compared with the control.

Previous studies have shown that THGP regulates the expression of the nuclear receptor NR4A2 in normal human fibroblasts under conditions of oxidative stress [28]. NR4A2 is also involved in inflammation; its expression increases during inflammatory responses, and it participates in negative feedback mechanisms to suppress inflammatory cytokine secretion [29]. Because adenosine signaling is upstream of NR4A2, we examined the effect of adenosine on NR4A2 expression after LPS stimulation. LPS increased NR4A2 expression 10-fold, whereas adenosine further increased NR4A2 expression by 16.4-fold (Figure 5a). Next, we evaluated NR4A2 expression after cotreatment with LPS and THGP. LPS increased NR4A2 expression by 4.4-fold, and THGP further increased NR4A2 expression to 7.7-fold (Figure 5b). Western blotting analysis also revealed that LPS stimulation increased NR4A2 protein levels by 1.25-fold, and THGP treatment significantly increased NR4A2 expression by 1.7-fold (Figure 5c,d). Finally, we investigated NR4A2 localization using immunofluorescence staining. LPS stimulation increased the proportion of cells with NR4A2 in their nucleus from 16% to 53% (Figure 5e,f). However, THGP treatment reduced the nuclear translocation of NR4A2 to 34%. These findings suggest that THGP regulates NR4A2 expression through adenosine signaling to suppress inflammation.

### 2.6. THGP Suppresses Inflammation by Regulating NR4A2 Expression

**Figure 6:**
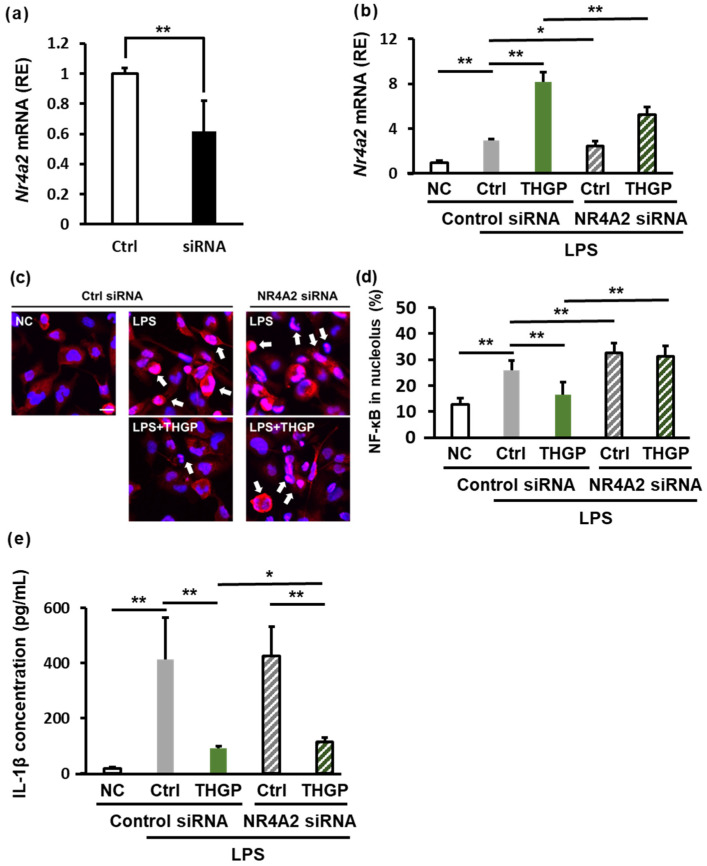
Analysis of the anti-inflammatory effects of THGP following NR4A2 knockdown. THP-1 cells were seeded and treated with 1 μM PMA for differentiation. After 24 h, the cells were treated with 5 mM THGP and transfected with siRNA. An additional 24 h later, 10 μg/mL LPS was added. The cells were then incubated for another 24 h, after which samples were collected. For IL-1β concentration measurement, the cells were stimulated with 1 mM ATP for 2 h. (**a**) qPCR verification of the NR4A2 knockdown efficiency. (**b**) Analysis of NR4A2 expression in cells treated with LPS (10 μg/mL), NR4A2 siRNA, and THGP (5 mM). (**c**) Images of immunofluorescence staining showing NF-κB localization (red) and nuclei (blue). The white arrows indicate NF-κB localized in the nucleus. Scale bar: 20 μm. (**d**) Quantification of the nuclear NF‒κB ratio. (**e**) ELISA measurement of IL-1β secretion from cells stimulated with LPS (10 μg/mL) and ATP (1 mM). The results are shown as the means ± SDs (*n* = 6). * *p* < 0.05 and ** *p* < 0.01 compared with the control.

To further investigate the role of NR4A2 in the anti-inflammatory effects of THGP, we used siRNA to knock down NR4A2 expression in THP-1 cells. Transfection of the NR4A2 siRNA reduced NR4A2 mRNA expression by approximately 40% (Figure 6a). RT‒PCR analysis revealed that LPS stimulation increased NR4A2 expression by 3-fold, and cotreatment with THGP increased NR4A2 expression by 8.2-fold. However, when the siRNA was transfected into the LPS + THGP group, NR4A2 expression decreased from 8.2-fold to 5.3-fold (Figure 6b). We also evaluated NF-κB localization after NR4A2 knockdown by immunofluorescence staining. LPS stimulation increased the proportion of cells with nuclear NF-κB localization from 13% to 26% (Figure 6c,d). THGP reduced this proportion to normal levels (16%). However, in cells that were transfected with NR4A2 siRNA, LPS-induced NF-κB nuclear localization increased to 32%, and the inhibitory effect of THGP on NF-κB nuclear translocation was abolished by NR4A2 knockdown. Finally, we assessed IL-1β secretion after LPS and ATP stimulation by ELISA (Figure 6e). Although THGP suppressed IL-1β secretion by 78%, this suppression was reduced to 73% in cells transfected with the NR4A2 siRNA compared with that in control cells. These results indicate that the anti-inflammatory effects of THGP are mediated by regulation of NR4A2 expression, although these effects are partially weakened by NR4A2 knockdown.

## 3. Discussion

In this study, we investigated the mechanism by which THGP suppresses inflammasome activation through the signal 1 pathway. Our findings revealed that THGP exerts anti-inflammatory effects by inhibiting IL-6 and TNF-α expression and IL-1β secretion through the formation of a complex with adenosine, which prevents adenosine degradation. Furthermore, this function may involve the suppression of NF-κB nuclear translocation through the adenosine–NR4A2 signaling pathway.

In our previous report, we demonstrated that THGP suppresses inflammasome activity by forming a complex with ATP [17]. However, when cells were stimulated with BzATP, which is a P2X7R agonist that cannot form complexes with THGP, we observed a slight inhibitory effect on inflammasome activation, although this effect was weaker than that observed after ATP stimulation. Based on these data, we hypothesized that the ATP-independent inhibitory effect on the inflammasome was mediated by the suppression of adenosine degradation.

Previous studies have shown that THGP forms complexes with substances containing *cis*-diol structures, such as ATP, adrenaline, and L-DOPA, and these complexes have various physiological effects on cells [17,18,19]. Additionally, THGP forms a complex with adenosine and inhibits its degradation by adenosine deaminase [25]. In this study, we performed cellular experiments and made the novel discovery that THGP exerts anti-inflammatory effects by inhibiting adenosine degradation through the formation of a complex.

Ge-132 (THGP) has been reported to suppress the expression of TNF-α, IL-1β, and IL-6 in mouse mammary cells treated with LPS [30]. The reported inhibition of p65 phosphorylation aligns with our current findings (Figure 1f). However, although previous studies reported the suppression of p38 phosphorylation, we did not observe this effect (Figure 1c). Given that Ge-132 has been reported to inhibit ERK and JNK phosphorylation, THGP may exert its effects through anti-inflammatory mechanisms beyond the p38–NF-κB signaling pathway [30].

Previously, we demonstrated that long-term treatment of RAW264.7 mouse macrophages with THGP led to their polarization toward the inflammatory M1 phenotype. This mechanism was suggested to be mediated by the effect of THGP on NF-κB nuclear translocation [14]. THGP-treated macrophages showed enhanced phagocytosis of foreign substances and cancer cells, along with increased expression of the IL-1β and TNF-α genes. In contrast, our current study revealed that THGP suppresses LPS-induced NF-κB nuclear translocation through adenosine signaling. These results suggest that THGP may activate macrophages under normal conditions but act as a suppressor under conditions characterized by excessive inflammation. Indeed, previous reports on Ge-132 have documented both immune-activating effects, such as Interferon-γ (IFN-γ) induction and enhanced macrophage and NK cell activity, which result in antitumor effects [31,32], as well as anti-inflammatory effects, including the suppression of rheumatic diseases partly via excessive TNF-α and IL-6 secretion [33]. These findings suggest that Ge-132 functions not only as an anti-inflammatory agent but also as an immunomodulator that enhances immunity under normal conditions while reducing inflammation under conditions of excessive inflammation.

When the ATP and adenosine levels in the THP-1 cell culture supernatant were measured, the results showed that THGP did not inhibit LPS-induced ATP release but suppressed adenosine degradation (Figure 3c,d). Although both ATP and adenosine possess *cis*-diol structures, suggesting that ATP degradation might also be inhibited, THGP did not suppress the degradation of ATP, unlike adenosine (Figure 3c). This difference can be attributed to the varying strengths of the complexes that are formed between THGP and these molecules because previous studies have reported weaker interactions between THGP and ATP than between THGP and adenosine [25].

Although THGP inhibited adenosine degradation, it promoted adenosine signaling (Figure 3). The active site of adenosine deaminase, which is an adenosine-degrading enzyme, requires ribose with a *cis*-diol structure [34]. In contrast, the adenosine receptor A2R binding site involves the adenine ring rather than the *cis*-diol region [35]. Because THGP forms complexes with adenosine at the *cis*-diol site, this difference in active sites explains why THGP can simultaneously inhibit adenosine degradation while allowing adenosine signaling to remain active.

In this study, the A2R antagonist reversed the inhibitory effect of THGP on IL-1β secretion (Figure 4e). Although previous research suggested that THGP-mediated inflammasome suppression was mediated by ATP complex formation, our findings revealed that this effect also depended on the inhibition of adenosine degradation.

Previously, we demonstrated that THGP suppresses IL-6 expression in normal human dermal fibroblasts under oxidative stress conditions [28]. The same study revealed that THGP strongly inhibits the oxidative stress-induced expression of NR4A2, which is a nuclear receptor that triggers cell death [36,37]. In contrast to previous findings, our current study revealed that THGP increased LPS-stimulated NR4A2 expression (Figure 5b). During inflammatory responses, LPS stimulation increases NR4A2 expression in macrophages, and NR4A2 knockdown promotes inflammation [38]. Similar findings have been reported in neuronal cells [39]. Additionally, NR4A2 has been shown to inhibit NF-κB activation [40]. NR4A2 appears to participate in a negative feedback loop, with its expression increasing during inflammation to suppress inflammatory responses [29]. Our study revealed that THGP further increased LPS-induced NR4A2 expression, suggesting that NF-κB expression was suppressed by increased NR4A2 expression. However, with respect to the cellular localization of NR4A2, whereas LPS stimulation induced the nuclear translocation of NR4A2, THGP treatment inhibited its nuclear translocation (Figure 5e). Previous studies have revealed varying effects of NR4A2 nuclear translocation on different cell types. In HT22 cells, hemin-induced inflammation triggers NR4A2 nuclear translocation, which is inhibited by the NR4A2 agonist amodiaquine, leading to reduced inflammation [41]. Conversely, in BV-2 microglia, LPS-induced NR4A2 nuclear translocation is enhanced by the NR4A2 agonist C-DIMs, reducing LPS-induced inflammation [40]. The relationship between NR4A2 localization and inflammation suppression requires further investigation.

NR4A2 knockdown with siRNA completely reversed the inhibitory effect of THGP on NF-κB nuclear translocation (Figure 6d). Similarly, blocking adenosine signaling with an adenosine receptor antagonist reversed the THGP-mediated suppression of NF-κB nuclear translocation (Figure 4b). These findings suggest that the THGP-mediated inhibition of NF-κB nuclear translocation depends on the adenosine–NR4A2 pathway. Although the adenosine receptor antagonist strongly suppressed the effects of THGP on IL-1β secretion, NR4A2 knockdown resulted in weaker suppression (Figure 4e and Figure 6d). However, compared with previous studies that used BzATP [17] and current findings obtained with adenosine antagonists, the NR4A2-mediated anti-inflammatory effects of THGP appear to be relatively minor. These findings suggest that the inhibitory effect of THGP on inflammasome activation is mediated primarily by two mechanisms: the formation of a complex with ATP and the suppression of adenosine degradation. Although NR4A2 plays a role in regulating inflammatory responses, additional key signaling pathways other than NR4A2 may be involved in IL-1β secretion.

Adenosine exerts anti-inflammatory effects and is implicated in various diseases. For example, adenosine is considered crucial for suppressing inflammation in rheumatic diseases [42]. THGP might serve as a potential therapeutic agent for rheumatic diseases by inhibiting adenosine degradation. Indeed, Ge-132, which is a THGP polymer, has been shown to ameliorate rheumatic diseases in both animal models and clinical trials [43,44]. Although this effect was attributed to immunomodulation, it might occur due to increased adenosine levels. Adenosine and A2A agonists have also been shown to suppress cancer progression, hypertension, and colitis [45,46]. THGP might, therefore, be effective in various adenosine-responsive conditions. Whereas A2A receptor agonists directly activate adenosine signaling [46], ADA inhibitors prevent adenosine degradation through enzyme inhibition [47]. In comparison, THGP uniquely promotes adenosine signaling by inhibiting ATP degradation through the formation of a complex with this molecule. This distinct mechanism suggests potential synergistic effects when combined with other adenosine signal activators.

**Figure 7:**
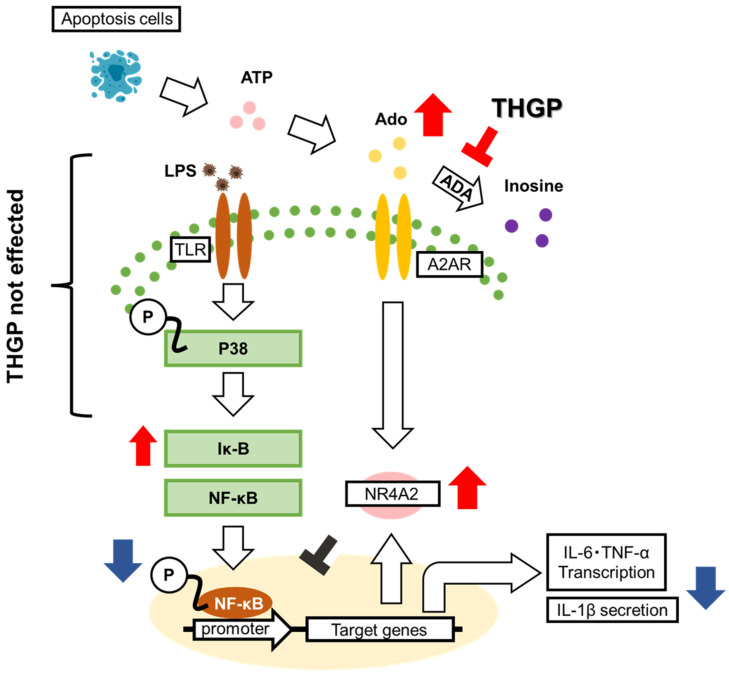
Schematic diagram of the mechanism of action of THGP against LPS-induced inflammation. THGP inhibits adenosine degradation by adenosine deaminase (ADA), leading to increased adenosine levels. Adenosine signaling activates NR4A2, which inhibits NF-κB nuclear translocation. Consequently, THGP suppresses IL-6 and TNF-α expression and IL-1β secretion. The blue arrows indicate a decrease due to THGP, while the red arrows indicate an increase due to THGP.

In conclusion, this study demonstrates that THGP exerts anti-inflammatory effects by forming a complex with adenosine, inhibiting its degradation, and activating the adenosine–NR4A2 pathway (Figure 7). THGP shows promise as a therapeutic and preventive agent for inflammatory diseases and various adenosine-related conditions. Future studies, including in vivo investigations, will examine these potential therapeutic applications.

## 4. Materials and Methods

### 4.1. Preparation of Reagents

Ge-132 was provided by Asai Germanium Research Institute Co., Ltd. (Kanagawa, Japan, Lot. 020X22A3). Ge-132 was dissolved in ultrapure water as THGP at a concentration of 500 mM, neutralized to pH 7.10 with NaOH (Wako Pure Chemical Industries Ltd., Osaka, Japan), and sterilized by filtration before use. For these experiments, THGP was added at a final concentration of 5 mM [17]. LPS from *Klebesiella pneumoniae* (Sigma-Aldrich Corp., St. Louis, MO, USA) was prepared at a concentration of 0.5 mg/mL in ultrapure water, sterilized by filtration, and stored at −30 °C. For these experiments, LPS was added at a final concentration of 10 μg/mL [48]. Adenosine or ATP (Tokyo Chemical Industry Co., Ltd., Tokyo, Japan) was prepared at concentrations of 10 mM or 100 mM in ultrapure water, sterilized by filtration, and stored at −30 °C. For these experiments, adenosine was added at a final concentration of 100 μM at the same time as LPS [17,49,50]. ATP was added at a final concentration of 1 mM 24 h after LPS stimulation. The A2AR receptor antagonist ZM24385 (Selleck Chemicals, Houston, TX, USA) was prepared at 10 mM in DMSO (Wako Pure Chemical Industries Ltd.) and stored at −30 °C. ZM24385 was added at a final concentration of 10 μM at the same time that LPS was added during these experiments. The siRNA (Invitrogen, Life Technologies Corp., Carlsbad, CA, USA) was dissolved in RNase-free water to a concentration of 10 μM and added at a final concentration of 50 nM at the same time as the LPS during these experiments.

### 4.2. Cell Culture

THP-1 cells were obtained from the Riken Cell Bank (Riken BRC, Ibaraki, Japan). The cells were cultured in RPMI medium supplemented with 10% FBS (Nissui Pharmaceutical Co., Ltd., Tokyo, Japan) at 37 °C in a humidified atmosphere with 5% CO_2_. Subculturing was performed every three days at a ratio of 1:5–10. For these experiments, THP-1 cells were differentiated into macrophage-like cells by adding 1 μM phorbol 12-myristate 13-acetate (PMA) (Sigma-Aldrich Corp.) at the time of seeding. The time course and the timing of drug treatments were shown in the Supplemental Figure.

### 4.3. Western Blotting

THP-1 cells were seeded in 6-cm dishes at a density of 2 × 10^6^ cells/well and cultured for 24 h in the presence of 1 μM PMA. THGP (5 mM) was then added, and the cells were cultured for another 24 h before the addition of LPS (10 μg/mL) and incubation for an additional 24 h. Proteins were extracted using RIPA buffer and quantified using the Bradford method (Bio-Rad Laboratories Inc., Hercules, CA, USA). Ten micrograms of protein was subjected to polyacrylamide gel electrophoresis. The primary antibodies used included anti-TLR4 (Diluted 1:200) (Santa Cruz Biotechnology, Inc., Santa Cruz, CA, USA), anti-p38 (Diluted 1:200) (Santa Cruz Biotechnology), anti-p-p38 (Diluted 1:1000) (Cell Signaling Technology, Danvers, MA, USA), anti-IκB (Cell Signaling Technology), anti-p65 (Santa Cruz Biotechnology), anti-p-p65 (Diluted 1:1000) (Cell Signaling Technology Inc.), anti-Nurr1/NR4A2 (Diluted 1:1000) (Santa Cruz Biotechnology), anti-adenosine deaminase (Diluted 1:200) (Santa Cruz Biotechnology), and anti-β-actin (Diluted 1:2000) (Abcam Ltd., Cambridge, UK) antibodies. The secondary antibodies (Diluted 1:5000) (Abcam) were used according to the corresponding primary antibodies. For phospho-specific antibodies, blocking and antibody dilution were performed with 5% bovine serum albumin (BSA) (Wako Pure Chemical Industries Ltd.), whereas for the other antibodies, 5% skim milk (Morinaga Milk Industry Co., Ltd., Tokyo, Japan) was used as the blocking and dilution buffer. β-Actin was used as an internal control for normalization during quantification.

### 4.4. Immunofluorescence Staining

THP-1 cells were seeded in a 6-well plate at a density of 1 × 10^6^ cells/well and cultured for 24 h in the presence of PMA (1 μM). THGP (5 mM) was then added, and the cells were cultured for another 24 h before the addition of LPS (10 μg/mL) and incubation for an additional 24 h. The cells were fixed with 4% paraformaldehyde in PBS (Wako Pure Chemical Industries Ltd.) and permeabilized with 0.2% Triton X-100 (Nacalai Tesque, Inc., Kyoto, Japan) for 10 min at room temperature. Blocking was performed with 1.5% BSA for 30 min at room temperature. Cells were incubated with the primary antibody overnight at 4 °C, followed by staining with a goat anti-mouse IgG H&L secondary antibody conjugated with Texas Red (Abcam) (Diluted 1:1000) for one hour at room temperature. The primary antibodies that were used included anti-p65 (Diluted 1:100) and anti-Nurr1/NR4A2 (Diluted 1:100) antibodies. Nuclei were stained with DAPI (Dojindo Laboratories, Kumamoto, Japan), and images were captured using a fluorescence microscope (BZ-X810, Keyence Co., Osaka, Japan).

### 4.5. RT‒PCR

THP-1 cells were seeded in a 6-well plate at a density of 1 × 10^6^ cells/well and cultured for 24 h in the presence of PMA (1 μM). THGP (5 mM) was then added, and the cells were cultured for another 24 h before the addition of LPS (10 μg/mL) and incubation for an additional 24 h. RNA was extracted using the ISOGEN II reagent (Nippon Gene Co., Ltd., Tokyo, Japan) according to the manufacturer’s protocol. One microgram of RNA was reverse-transcribed using SuperScript III Reverse Transcriptase (Invitrogen) at 50 °C for one hour, followed by heat inactivation at 95 °C for five minutes. Real-time PCR was performed using TB Green Premix Ex Taq II (Tli RNaseH Plus) reagent (Takara Bio Inc., Otsu, Japan) under conditions of denaturation at 95 °C for five seconds and annealing/extension at 60 °C for thirty seconds over forty cycles. RPS18 served as an internal control for normalization. The primers used for RT-PCR are shown in Table 1.

### 4.6. Measurement of ATP Levels

THP-1 cells were seeded in a 96-well plate at a density of 2 × 10^4^ cells/well and cultured for 24 h in the presence of 1 μM PMA. Then, THGP (5 mM) was added, and the cells were cultured for another 24 h. Subsequently, LPS (10 μg/mL) was added, and the cells were cultured for an additional 24 h. The ATP levels in the culture supernatant were measured using a CellTiter Glo 2.0 assay kit (Promega Corp., Fitchburg, WI, USA) according to the manufacturer’s protocol.

### 4.7. Measurement of Adenosine Levels

THP-1 cells were seeded in a 24-well plate at a density of 3 × 10^5^ cells/well and cultured for 24 h in the presence of 1 μM PMA. THGP (5 mM) was then added, and the cells were cultured for another 24 h. After the culture supernatant was removed, THGP (5 mM) and LPS (10 μg/mL) were added, and the cells were cultured for an additional 24 h. The culture supernatant was collected, and adenosine levels were measured using an adenosine assay kit (Cell Biolabs, Inc., San Diego, CA, USA) according to the manufacturer’s protocol.

### 4.8. Measurement of Inosine Levels

THP-1 cells were seeded in a 10 cm dish at a density of 1 × 10^7^ cells/100 mm dish and cultured for 24 h in the presence of 1 μM PMA. THGP (5 mM) was then added, and the cells were cultured for another 24 h. The total number of THP-1 cells was adjusted to 3 × 10^7^ cells, and membrane proteins were extracted using the Trident Membrane Protein Extraction Kit (GeneTex, Irvine, CA, USA). The extracted proteins (10 μg) were incubated with adenosine (200 μM) in Tris buffer (100 mM Tris-HCl, 0.5 mM CaCl_2_, 0.5 mM MgCl_2_, pH 7.5) at 37 °C for four hours. The reaction was terminated by heating at 100 °C for 15 min. The amount of inosine in the reaction mixture was measured via HPLC under the following conditions: column—Inertsil^®^ ODS-SP column (particle size—5 µm; dimensions—4.0 mm × 150 mm; GL Sciences, Tokyo, Japan); mobile phase—12.5 mM tetra-n-butylammonium hydroxide in 20 mM NH4H2PO4/methanol = 75:25; flow rate—1.2 mL/min; column temperature—35 °C; and detection wavelength—260 nm.

### 4.9. ^1^H-NMR Analysis

The interaction between THGP and adenosine or their mixtures was analyzed using ^1^H-NMR spectroscopy with a Mercury Plus spectrometer operating at 300 MHz (Agilent Technologies Inc., Santa Clara, CA, USA). The samples were prepared at a concentration of 10 mM with the pH adjusted to 7.0–7.5 and mixed at a molar ratio of 1:1. Deuterium oxide (Kanto Chemical Co., Inc., Tokyo, Japan) was used as the solvent for all the samples. The residual light water in deuterium oxide (HOD: offset at 4.80 ppm) served as an internal chemical shift standard. Measurements were performed at a temperature of 30 °C, with an accumulation count of 256 scans.

### 4.10. ELISA for IL-1β Quantification

THP-1 cells were seeded in a 24-well plate at a density of 1 × 10^5^ cells/well and cultured for 24 h in the presence of PMA (1 μM). THGP (5 mM) was then added, and the cells were cultured for another 24 h before the addition of LPS (10 μg/mL) and incubation for an additional 24 h. Subsequently, ATP (1 mM) was added, and the cells were incubated for two hours before the culture supernatant was collected. IL-1β levels in the supernatant were quantified using a human IL-1β ELISA kit (Diaclone SAS, Besançon, France) according to the manufacturer’s instructions.

### 4.11. Measurement of Inflammatory Markers After A2R Inhibitor Treatment

THP-1 cells were seeded and cultured for 24 h in the presence of PMA (1 μM). THGP (5 mM) was then added, and the cells were cultured for another 24 h before THGP (5 mM), LPS (10 μg/mL), and the A2R inhibitor ZM24385 (10 μM) were added. The effects of the ZM24385 treatment on inflammatory markers were evaluated by assessing NF-κB nuclear translocation via immunofluorescence staining (Section 4.4), cytokine mRNA expression via real-time RT‒PCR (Section 4.5), and IL-1β secretion via ELISA (Section 4.10).

### 4.12. Measurement of Inflammatory Markers After NR4A2 Knockdown Using an siRNA

Silencer Select siRNA targeting NR4A2 (#s9785; Thermo Fisher Scientific Inc., Madison, MA, USA) or Negative Control No.1 siRNA (Thermo Fisher Scientific) was used for the knockdown experiments. siRNA and Lipofectamine RNAiMAX Transfection Reagent (Thermo Fisher Scientific) were each diluted in Opti-MEM™ I Reduced Serum Medium (Thermo Fisher Scientific), incubated at room temperature for five minutes, and then combined before being added to cells at final concentrations of siRNA (50 nM) siRNA and Lipofectamine RNAiMAX reagent (2 μL/mL). The effects of NR4A2 knockdown on inflammatory markers were assessed by evaluating NF-κB nuclear translocation via immunofluorescence staining (Section 4.4), cytokine mRNA expression via real-time RT‒PCR (Section 4.5), and IL-1β secretion via ELISA (Section 4.10).

### 4.13. Statistical Analysis

Statistical analyses were performed using Statcel4 software ver.3 (OMS Publishing Co., Ltd., Tokyo, Japan). A Student’s *t*-test was used to compare two groups, whereas one-way ANOVA followed by the Tukey‒Kramer multiple comparison test was used to evaluate differences among three or more groups. A *p*-value < 0.05 was considered to indicate statistical significance. All the graphs present the mean values ± standard deviations across replicates.

## Figures and Tables

**Table 1:** Primer list.

Target mRNA	Forward Primer Sequence (5′→3′) Reverse Primer Sequence (5′→3′)	Amplicon Size (bp)	Gene Reference
***Tnf-α***	TCAGCCTCTTCTCCTTCCTG GGCTACAGGCTTGTCACTCG	173	NM_000594.4
***Il-6***	GGATTCAATGAGGAGACTTGC GTTGGGTCAGGGGTGGTTAT	197	NM_001318095.2
***Nr4a2***	CTAACCTGCAGGCAGAACCTGAA ACATTTGTCTGAACTGCAACCA	197	NM_006186.4
***Rps18***	TTTGCGAGTACTCAACACCAACATC GAGCATATCTTCGGCCCACAC	89	NM_022551.3

## Data Availability

The data that support the findings of this study are available from the corresponding author upon reasonable request.

## Supplementary Materials

The supporting information can be downloaded at https://www.mdpi.com/article/10.3390/ijms26062449/s1.
