# Supplementary material for: The Organogermanium Compound 3-(trihydroxygermyl)propanoic Acid Exerts Anti-Inflammatory Effects via Adenosine-NR4A2 Signaling
Source: Int J Mol Sci. 2025 Mar 9;26(6):2449. doi: 10.3390/ijms26062449 (PMC11941763; doi:10.3390/ijms26062449)
Supplement: Supplementary file 1 [file ijms-26-02449-s001.zip › ijms-3512077-supplementary.pdf]

Supplemental Figure

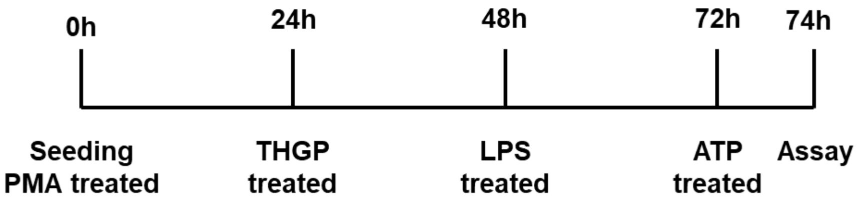

Experimental schedule for treatments in THP-1 cells.

THP-1 cells were seeded and treated with 1 μM PMA at 0 hours for differentiation. After 24 hours, the cells were washed with PBS (-) and treated with 5 mM THGP. After an additional 24 hours (48 hours from the start), 10 μg/mL LPS was added. The cells were then incubated for another 24 hours (72 hours from the start), after which samples were collected for PCR, Western blot, and immunofluorescence assays. For IL-1β concentration measurement, 1 mM ATP was added at 72 hours, and the culture supernatant was collected 2 hours later (74 hours from the start). Additionally, specific treatments were applied in each figure as follows: Figure 2: 100 μM adenosine was added at the same time as LPS (48h). Figure 4: 10 μM ZM24385 was added at the same time as LPS (48h). Figure 6: siRNA was transfected at the time of THGP treatment (24h).
